# Supplementary material for: Dynamics of Circulating CD14/CD16 Monocyte Subsets in Obstructive Sleep Apnea Syndrome Patients upon Hypoglossal Nerve Stimulation
Source: Biomedicines. 2022 Aug 9;10(8):1925. doi: 10.3390/biomedicines10081925 (PMC9405940; doi:10.3390/biomedicines10081925)
Supplement: Supplementary file 1 [file biomedicines-10-01925-s001.zip › biomedicines-1816491-supplementary.pdf]

biomedicines

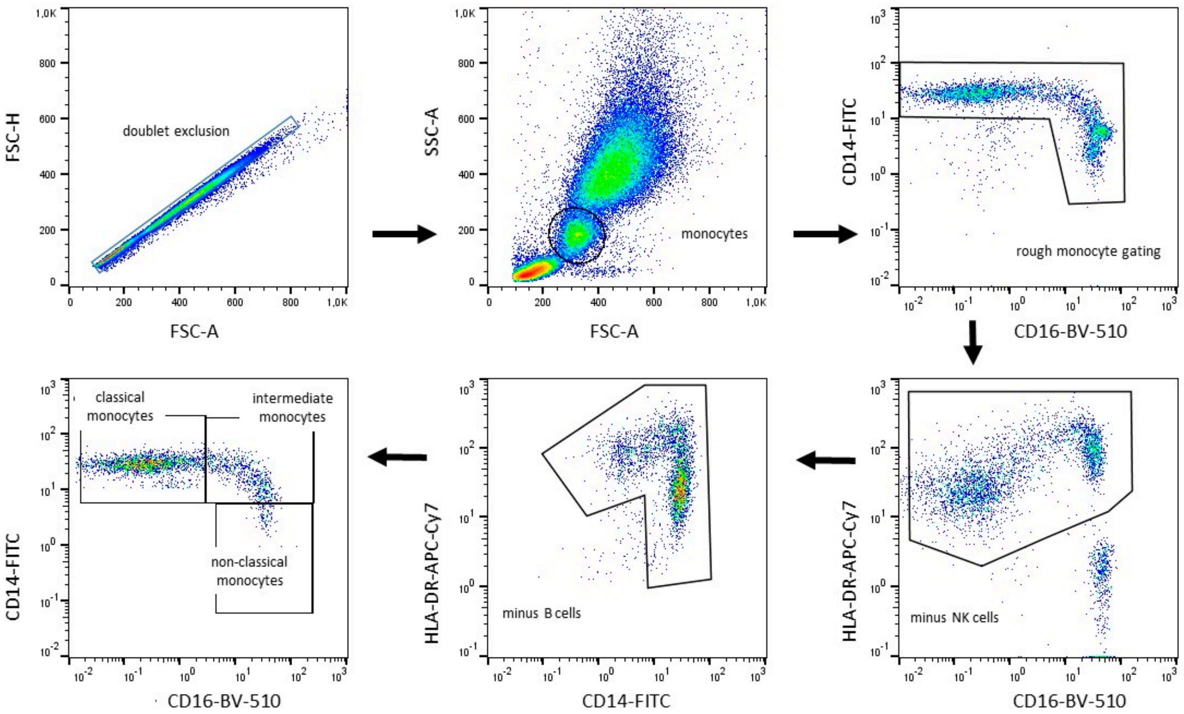

Figure S1. Flow cytometric identification of monocyte subsets. After doublet exclusion of CD45+ leucocytes, a rough gating of monocytes was first carried out by their forward scatter (FSC) and side scatter (SSC) characteristics and further by their CD14 and CD16 expression. NK cells and neutrophil granulocytes were excluded by their missing HLS-DR expression and remaining B cells by their missing CD14 expression. Finally, monocyte subsets were subdivided into $CD14^{++}CD16^{-}$ "classical" monocytes, $CD14^{+}CD16^{+}$ "intermediate" and $CD14^{dim+}CD16^{+}$ "non-classical" monocytes.
